# Impact of mitochondrial DNA mutations in multiple myeloma

**DOI:** 10.1038/s41408-020-0315-4

**Published:** 2020-05-01

**Authors:** Phuc H. Hoang, Alex J. Cornish, Daniel Chubb, Graham Jackson, Martin Kaiser, Richard S. Houlston

**Affiliations:** 1 Division of Genetics and Epidemiology, The Institute of Cancer Research, London, UK; 2 Division of Molecular Pathology, The Institute of Cancer Research, London, UK; 3 Department of Haematology, University of Newcastle, Newcastle Upon Tyne, UK

**Keywords:** Cancer genomics, Myeloma

Dear Editor,

Most cancers have altered metabolism with increased uptake of glucose (i.e. the “Warburg effect”) attributed to defective mitochondria^1^. In addition, mitochondria are associated with multiple key processes linked to tumourigenesis including apoptosis, cell cycle, cell growth, and signalling^2^. Multiple myeloma (MM) is essentially an incurable haematological malignancy, with most patients developing resistance to treatment and eventually dying from relapse. Recent studies have proposed mitochondria dysfunction is important in defining chemotherapy resistance and disease progression in MM^3,4^. Such an assertion is supported by pre-clinical studies, which have suggested agents targeting mitochondria in relapsed MM can improve patient outcome^5,6^. Thus far, the spectrum of mitochondrial DNA (mtDNA) mutations and their functional implications in MM have not however been well characterised, partly due to limited sample size and whole-exome sequencing depth^7^. Furthermore, the paucity of MM representation in pan-cancer analyses^7^ has not allowed an appraisal of MM-specific mitochondrial mutations. By analysing whole-genome sequencing (WGS) data from the Myeloma XI trial, we have sought to address these shortcomings, characterising the somatic mutation landscape, mutation selection at relapse, nuclear genome integration, and copy number of MM mitochondria.

To investigate mtDNA mutations in MM, we analysed WGS data on 80 matched tumour and normal samples from newly diagnosed patients, of which 25 also had matched relapsed tumours. Owing to high cellular copy number of mtDNA genomes, we obtained far greater mtDNA genome coverage (normals: median 2149×, range 1015×–7777×; primary tumours: median 7836×, range 2376×–7938×; relapsed tumours: median 7826×, range 4678×–7929×) compared to the nuclear genome (Supplementary Table 1).

We identified 210 mtDNA single nucleotide variants (SNVs) in the 80 primary tumours (median 3 SNVs/tumour). These showed strong replicative strand bias, predominantly C>T on heavy strand and T>C on light strand (Supplementary Fig. 1), which has previously been ascribed to replication-coupled process partly due to the lack of transcriptional strand bias^7^. Examining the sequence context of mutations revealed the contribution of defective transcription-coupled DNA repair COSMIC signatures 12 (16%), 21 (15%), 23 (11%), and 26 (48%) (Fig. 1a). We observed transcriptional strand bias across all genes (Fig. 1b), with the strongest signal for C>T, where transcribed strand are more frequently repaired^8^. The weaker transcriptional strand bias for T>C is likely due to the neutralising effects from COSMIC signatures with opposing transcriptional strand biases (Supplementary Fig. 2). To validate these observations, we repeated the analysis of mtDNA mutational spectra using WGS data from 850 newly diagnosed MM^9,10^ generated by The Relating Clinical Outcomes in Multiple Myeloma to Personal Assessment of Genetic Profile Study (CoMMpass; tumour and normal sample median read depth of 869× and 661×, respectively). The mutational spectra and strand biases observed in the Myeloma XI samples were also apparent in CoMMpass (Supplementary Fig. 3). Transcriptional strand biases in the CoMMpass samples persist when considering the 22 tRNA genes (14 light strand and 8 heavy strand) separately (Supplementary Fig. 3d). Collectively, these findings are consistent with the contribution of transcription-coupled DNA repair defects in MM mtDNA.

**Fig. 1:**
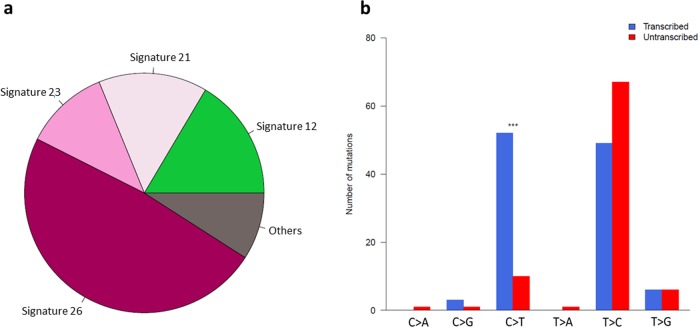
Mutational signatures in mitochondrial DNA of 80 primary tumours from Myeloma XI trial. **a** Contribution of COSMIC mutational signatures extracted by deconstructSigs. **b** Transcriptional strand biases across all mitochondrial genes. Significant difference in strand bias was assessed by proportion tests. **P < 0.01, ***P < 0.001.

Within the 80 Myeloma XI trial samples, 14/210 (6%) of somatic mutations were identified as being pathogenic (Supplementary Table 2); a number being associated with established diseases^11^ including m.4136A>G (Leper’s optic atrophy), m.9185T>C (Charcot–Marie–Tooth disease, Leigh syndrome, complex V deficiency), m.15246G>A (development delay, hearing impairment, macrocephalus), and m.15287T>C (familial breast cancer). As mitochondrial disease is rare in the general population (around 1 in 5000)^12^, it is likely these mutations have a direct effect on gene function.

We did not observe significant difference in mtDNA somatic mutational burden between MM subtypes, or between primary and relapse tumours (Supplementary Fig. 4). Most germline variants are homoplasmic, whereas somatic variants are more variable in their heteroplasmic level (*P* < 2.2 × 10^−16^, Wilcoxon rank-sum test) (Supplementary Fig. 5). The majority of germline mutations are located outside protein-coding regions or synonymous mutations, with no loss-of-function (i.e. truncating) variants detected (Fig. 2a). In contrast, somatic mutations are more enriched for missense and truncating variants (*P* < 2.2 × 10^−16^) (Fig. 2a), suggesting germline and somatic variants are under different selection constraints. The most frequently disrupted mtDNA coding genes by non-synonymous somatic mutations include *MT-ND5* (29% of primary tumours), *MT-ND4* (24%), *MT-CO1* (20%), and *MT-ND1* (15%) (Supplementary Table 3).

**Fig. 2:**
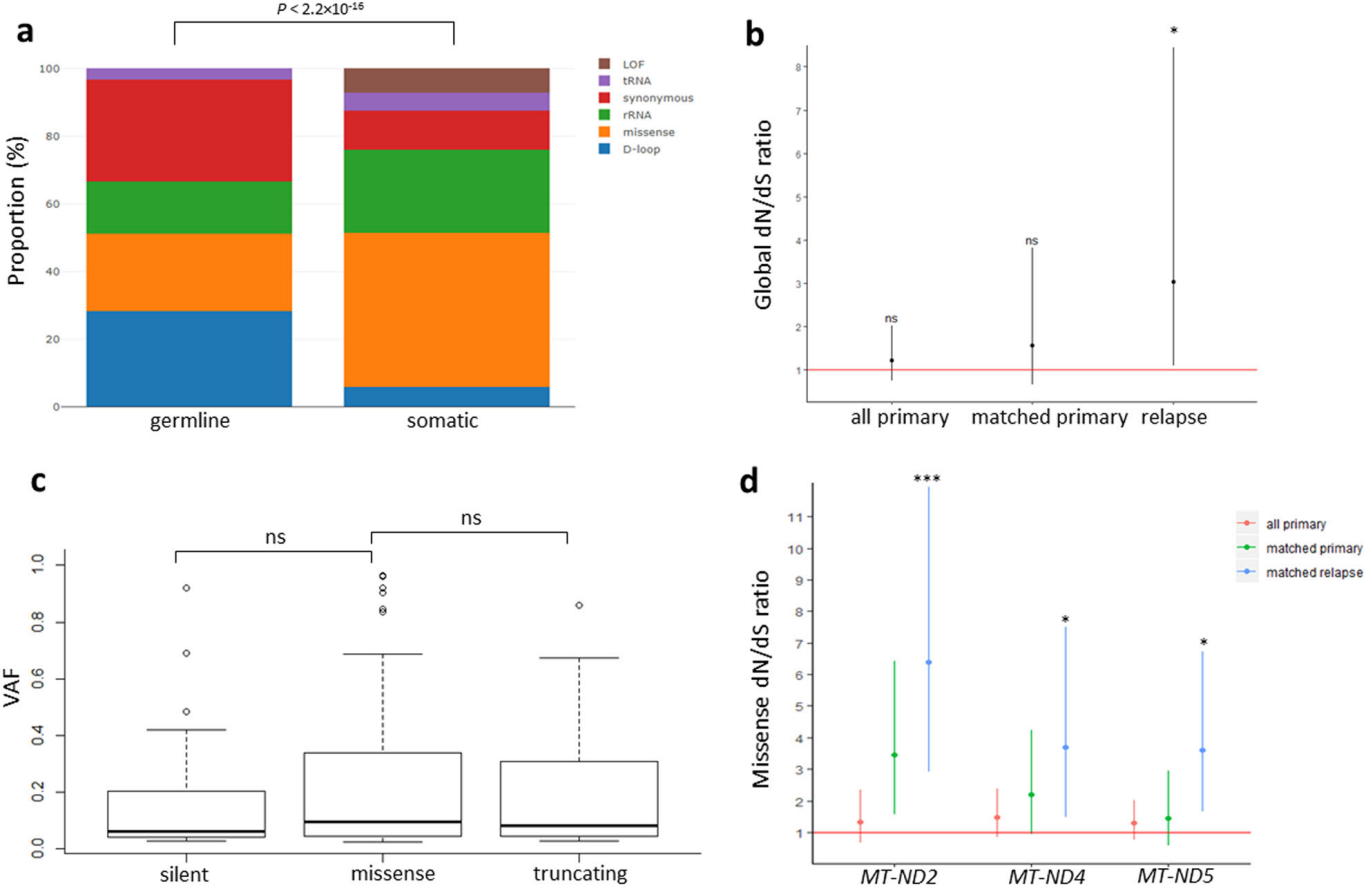
Selection of mtDNA somatic mutations in primary and relapsed multiple myeloma tumours. **a** Proportion of mutation type in mitochondrial germline and somatic mutations. Difference in mutation-type contribution was assessed by *χ*^2^ test. **b** Global d*N*/d*S* ratio for all 80 primary tumours, 25 matched primary tumours, and 25 relapsed tumours. *P < 0.05. Vertical lines depict 95% confidence intervals. **c** Heteroplasmic level comparison between silent (*n* = 26), missense (*n* = 102), and truncating mutations (*n* = 23) in 80 primary tumours. Whisker bars extend within ± 1.5× interquartile range. **d** Missense d*N*/d*S* ratio for *MT-ND2*, *MT-ND4*, and *MT-ND5* suggest positive selection of missense mutations in these genes at relapse. Vertical lines depict 95% confidence intervals. *Q < 0.05, ***Q < 0.001. LOF loss-of-function (i.e. truncating mutations), VAF variant allele frequency, ns not significant.

The d*N*/d*S* ratio provided no evidence of positive or negative selection for somatic mutations in primary tumours (d*N*/d*S* = 1.24, 95% CI: 0.76–2.03; *P* = 0.39) (Fig. 2b), consistent with the observation that missense and truncating mutations do not have significantly different heteroplasmic levels compared to silent mutations (Fig. 2c). However, non-synonymous mutations were positively selected at relapse (d*N*/d*S* ratio 3.01, 95% CI: 1.09–8.25; *P* = 0.033) (Fig. 2b), in concordance with significant increase in homoplasmy of non-synonymous mutations at relapse (Supplementary Fig. 6). Notably, missense mutations in mitochondrial genes composed of the NADH dehydrogenase complex (*MT-ND2*, *MT-ND4*, and *MT-ND5*), feature a higher than expected rate of missense mutations (i.e. positively selected) at relapse (*Q* < 0.05) (Fig. 2d), with non-synonymous mutations in *MT-ND5* and *MT-CO3* being most frequently acquired at relapse (Supplementary Table 4). These findings imply potential survival advantage rendered through disruption of these genes.

We next sought to examine the effects of mtDNA copy numbers and somatic transfer in MM. We did not find significant difference between mtDNA copy number of tumours and their matched normal, relapsed tumours versus primary tumours, or between high- and low-risk MM subtypes (Supplementary Fig. 7). The results therefore do not support pathogenic and prognostic contribution of mtDNA copy number in MM.

We observed 11/80 primary tumours and 6/25 relapsed tumours positive for somatic transfer of mtDNA to nuclear DNA (Supplementary Table 5). Transfer breakpoints disrupt open reading frames of known oncogenes including *CENPP*, *FOXK1*, *MGAT5*, *ST8SIA1*, and *RAB4A*, suggesting a potential role in MM tumourigenesis.

We present here the mtDNA mutational spectrum of MM, the potential underlying mutational processes, and mechanisms in which they could contribute to MM development. We observed transcriptional strand bias of somatic mutations, suggesting transcription-coupled DNA repair defects as one of the main contributing mutational processes in MM mtDNA. This observation is consistent with mitochondria having reduced DNA repair pathways^13^. A larger cohort would be required to unambiguously deconvolve the contribution of each mutational signature at higher nucleotide context resolution. As different defective transcription-coupled DNA repair processes have opposing transcriptional strand biases^8^ and their contribution are varied across tumour types, the transcriptional strand bias might have been neutralised in a previous pan-cancer analysis^7^.

We did not find evidence supporting either negative or positive selection in primary tumours. However, our results do support positive selection at relapse, potentially providing survival and resistance advantage for MM tumours. Consistent with this, we observed significant d*N*/d*S* ratio for missense mutations for genes comprising complex I (*MT-ND2*, *MT-ND4*, and *MT-ND5*) and mutations disrupting *MT-ND5* and *MT-CO3* (cytochrome *c* oxidase) are frequently acquired at relapse. Functional studies have suggested mutations impacting mitochondrial genes can recapitulate the Warburg effect and provide an alternative mechanism for tumour growth^14^. Although mtDNA copy numbers do not have pathogenic or prognostic implication in MM, mitochondria-nuclear genome integration could potentially contribute to tumourigenesis through disruption of oncogenic genes (e.g. *CENPP*, *FOXK1*, *MGAT5*, *ST8SIA1*, and *RAB4A*).

In summary, our study provides evidence to support mitochondrial mutations disrupting electron transport chain, providing potential growth and resistance at relapse MM. Further studies are required to examine the clinical value of mitochondrial mutations as biomarkers, and explore the therapeutic potential of targeting dysregulated metabolism in MM.

## Supplementary information

Supplementary information

Supplementary Figures

Supplementary Table
