# Supplementary material for: Impact of mitochondrial DNA mutations in multiple myeloma
Source: Blood Cancer J. 2020 May 1;10(5):46. doi: 10.1038/s41408-020-0315-4 (PMC7195394; doi:10.1038/s41408-020-0315-4)
Supplement: Supplementary file 1 — Supplementary information [file 41408_2020_315_MOESM1_ESM.docx]

**METHODS**

**Samples**

Bone marrow aspirates and blood samples were obtained from patients with newly diagnosed MM being treated according to the UK National Cancer Research Institute Myeloma XI trial protocol^1^. Tumour DNAs were extracted from plasma cells selected and sorted using CD138 microbeads as described previously^2^. Germline DNA was derived from matched blood samples. Tumour *IGH*-translocation status was determined using multiplexed real-time PCR^3^. Clinical data and informed consent was obtained from all patients. Ethical approval for the study was obtained by the Oxfordshire Research Ethics Committee (MREC 17/09/09, ISRCTN49407852).

Sequencing libraries were prepared using Illumina SeqLab specific TruSeq Nano High Throughput library preparation kit (Illumina Inc, San Diego, CA 92122 USA) and paired end sequencing was conducted using Illumina HiSeqX technology. Raw WGS sequencing data were quality checked using FastQC (v.0.11.4) and aligned using the Burrows-Wheeler Alignment tool^4^ (BWA v0.7.13) to the human genome hg38 assembly and human mtDNA revised Cambridge Reference sequence (rCRS)^5^ using default parameters.

**Variant calling**

Mitochondrial somatic and germline variants from 80 matched tumour-normal pairs were called using MuTect2 (v4.0.3.0)^6^ according to best practices, using The Genome Aggregation Database (gnomAD)^7^ file in GRCh38 provided as part of the GATK resource. Additional somatic variants called from 850 WGS tumour-normal pairs, generated as part of the Multiple Myeloma Research Foundation (MMRF) CoMMpass Study (release IA10)^8, 9^, were used to independently validate mutational spectrum and strand biases. Somatic variants were filtered for cross-sample contamination, oxidation artefact, alternative allele frequency > 2.5%, base quality score > 20, mapping quality score > 20, and at least one alternative read in each strand direction^10^. We also excluded variants in known false positive regions (rCRS 302-315, rCRS 513 – 525, and 3105-3110)^11^. Recurrent germline variants (present in >10% of samples) were further removed^10^ if they are not reported in MitoMap database (<https://mitomap.org/MITOMAP>)^12^. All variants were annotated for functional and pathogenic implications using Mitochondrial Disease Sequence Data Resource (MSeqDR, <https://mseqdr.org/>)^13^. Functional implication of tRNA variants was evaluated using MitoTIP^14^, where likely pathogenic variants are those with MitoTIP score >16.25 and >75% quartile of pathogenicity score database.

**Strand bias and mutational signatures analysis**

Analysis of replication and transcriptional strand bias was performed as previously described^11^ for Myeloma XI (80 primary tumours) and CoMMpass (850 primary tumours)^8^ cohorts independently. Briefly, substitution rates for each of the 96 trinucleotide context on L and H strands were calculated and normalised for trinucleotide context^8^. To examine replication and transcriptional strand biases, we considered 12 substitution classes: 6 possible base substitution × 2 strands (H/L strand or transcribed/non-transcribed strand)^15^. We included all substitutions for replication bias analysis, while transcriptional strand bias was considered for substitutions residing in mtDNA genes (13 protein-coding, 22 tRNA, and 2 rRNA genes). The proportion test was used to determine significant difference in strand biases.

Signature fitting of all primary somatic mutations against with 30 Catalogue of Somatic Mutations in Cancer (COSMIC) signatures (<https://cancer.sanger.ac.uk/cosmic/signatures/>) were carried out using deconstructSigs^16^ with default settings, considering mitochondrial mutations across all tumours as performed previously^11^ for Myeloma XI and CoMMpass cohorts independently*.*

**dN/dS**

dN/dS values for somatic variants were calculated globally and across 13 mitochondrial coding genes using dNdScv R package with default parameters^17^. To minimise the effect of extreme replication bias^11^, *MT-ND6* on H strand was excluded when estimating global dN/dS values. The Benjamini-Hochberg FDR procedure was used to adjust for multiple hypothesis testing with coding genes with significance thresholded at *Q* < 0.05.

**Mitochondrial copy number and heteroplasmy estimation**

Mitochondrial copy number were estimated using fastMitoCalc with default parameters^18^, with tumour mitochondrial DNA copy number (${CN}_{tumour}$) corrected for tumour ploidy ($n_{tumour}$) and tumour purity ($\rho$) using the following formula^19^:

$${CN}_{tumour}=\frac{mtDNA mean coverage depth}{autosomal DNA mean coverage depth}(\rho\times n_{tumour}+(1- \rho)\times2)$$

Tumour ploidy and purity were estimated by Battenberg^20^, with purity compared and corrected using Ccube^21^. When comparing variant allele frequency (VAFs) between shared primary and relapse mutations of patient $i$ (${VAF}^{i}$), VAF were normalised for purity as:

$${VAF}_{relapse normalised}^{i}=\frac{r_{alt}}{{(r}_{alt}+ r_{ref})}\times\frac{\rho_{primary}^{i}}{\rho_{relapse}^{i}}$$

where $r_{alt}$ , $r_{ref}$are number of alternative reads and reference reads respectively.

**Somatic mitochondrial transfer**

Identification of mitochondria somatic nuclear transfer integration to nuclear genome was performed using MitoSeek^22^. To minimise false positives, we only considered events supported by at least 5 reads^23^ and excluded events with the same breakpoints present in ≥ 3 samples.

**Data availability**

Raw WGS data from Myeloma UK XI trial generated as part of this study and CoMMpass study can be accessed through EGA (accession code EGAS00001003926) and dbGaP (accession code phs000748.v4.p3) respectively.

**REFERENCES**

1. Jackson, G.H. *et al.* Lenalidomide maintenance versus observation for patients with newly diagnosed multiple myeloma (Myeloma XI): a multicentre, open-label, randomised, phase 3 trial. *Lancet Oncol* **20**, 57-73 (2019).

2. Walker, B.A. *et al.* A compendium of myeloma-associated chromosomal copy number abnormalities and their prognostic value. *Blood* **116**, e56-65 (2010).

3. Kaiser, M.F. *et al.* A TC classification-based predictor for multiple myeloma using multiplexed real-time quantitative PCR. *Leukemia* **27**, 1754-1757 (2013).

4. Langmead, B., Trapnell, C., Pop, M. & Salzberg, S.L. Ultrafast and memory-efficient alignment of short DNA sequences to the human genome. *Genome Biol* **10**, R25 (2009).

5. Andrews, R.M. *et al.* Reanalysis and revision of the Cambridge reference sequence for human mitochondrial DNA. *Nat Genet* **23**, 147 (1999).

6. Costello, M. *et al.* Discovery and characterization of artifactual mutations in deep coverage targeted capture sequencing data due to oxidative DNA damage during sample preparation. *Nucleic Acids Res* **41**, e67 (2013).

7. Karczewski, K.J. *et al.* Variation across 141,456 human exomes and genomes reveals the spectrum of loss-of-function intolerance across human protein-coding genes. *bioRxiv*, 531210 (2019).

8. Hoang, P.H., Cornish, A.J., Dobbins, S.E., Kaiser, M. & Houlston, R.S. Mutational processes contributing to the development of multiple myeloma. *Blood Cancer Journal* **9**, 60 (2019).

9. Hoang, P.H. *et al.* Whole-genome sequencing of multiple myeloma reveals oncogenic pathways are targeted somatically through multiple mechanisms. *Leukemia* (2018).

10. Triska, P. *et al.* Landscape of Germline and Somatic Mitochondrial DNA Mutations in Pediatric Malignancies. *Cancer Res* **79**, 1318-1330 (2019).

11. Ju, Y.S. *et al.* Origins and functional consequences of somatic mitochondrial DNA mutations in human cancer. *Elife* **3** (2014).

12. Lott, M.T. *et al.* mtDNA Variation and Analysis Using Mitomap and Mitomaster. *Curr Protoc Bioinformatics* **44**, 1 23 21-26 (2013).

13. Shen, L. *et al.* MSeqDR: A Centralized Knowledge Repository and Bioinformatics Web Resource to Facilitate Genomic Investigations in Mitochondrial Disease. *Hum Mutat* **37**, 540-548 (2016).

14. Sonney, S. *et al.* Predicting the pathogenicity of novel variants in mitochondrial tRNA with MitoTIP. *PLoS Comput Biol* **13**, e1005867 (2017).

15. Adalsteinsson, V.A. *et al.* Scalable whole-exome sequencing of cell-free DNA reveals high concordance with metastatic tumors. *Nat Commun* **8**, 1324 (2017).

16. Rosenthal, R., McGranahan, N., Herrero, J., Taylor, B.S. & Swanton, C. DeconstructSigs: delineating mutational processes in single tumors distinguishes DNA repair deficiencies and patterns of carcinoma evolution. *Genome Biol* **17**, 31 (2016).

17. Martincorena, I. *et al.* Universal Patterns of Selection in Cancer and Somatic Tissues. *Cell* **171**, 1029-1041 e1021 (2017).

18. Qian, Y. *et al.* fastMitoCalc: an ultra-fast program to estimate mitochondrial DNA copy number from whole-genome sequences. *Bioinformatics* **33**, 1399-1401 (2017).

19. Yuan, Y. *et al.* Comprehensive Molecular Characterization of Mitochondrial Genomes in Human Cancers. *bioRxiv*, 161356 (2017).

20. Nik-Zainal, S. *et al.* The life history of 21 breast cancers. *Cell* **149**, 994-1007 (2012).

21. Yuan, K., Macintyre, G., Liu, W. & Markowetz, F. Ccube: A fast and robust method for estimating cancer cell fractions. *bioRxiv*, 484402 (2018).

22. Guo, Y., Li, J., Li, C.-I., Shyr, Y. & Samuels, D.C. MitoSeek: extracting mitochondria information and performing high-throughput mitochondria sequencing analysis. *Bioinformatics* **29**, 1210-1211 (2013).

23. Ju, Y.S. *et al.* Frequent somatic transfer of mitochondrial DNA into the nuclear genome of human cancer cells. *Genome Res* **25**, 814-824 (2015).
