# Supplementary material for: Impact of mitochondrial DNA mutations in multiple myeloma
Source: Blood Cancer J. 2020 May 1;10(5):46. doi: 10.1038/s41408-020-0315-4 (PMC7195394; doi:10.1038/s41408-020-0315-4)
Supplement: Supplementary file 2 — Supplementary Figures [file 41408_2020_315_MOESM2_ESM.pdf]

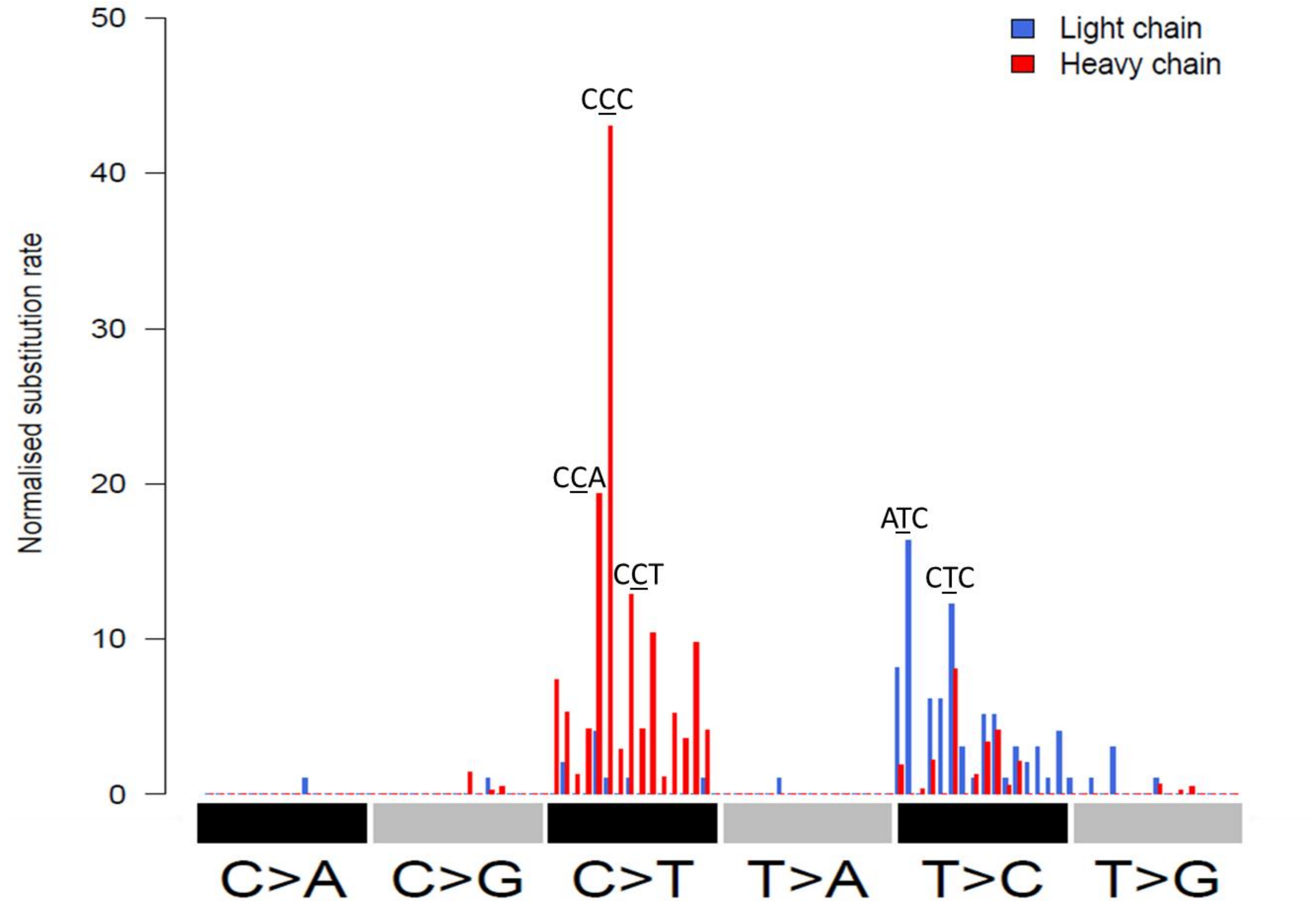

Supplementary Figure 1: Mutational patterns by 96 trinucleotide context across 80 primary tumours from Myeloma XI trial. Substitution rate is normalised for trinucleotide context difference between mitochondrial light and heavy chains.

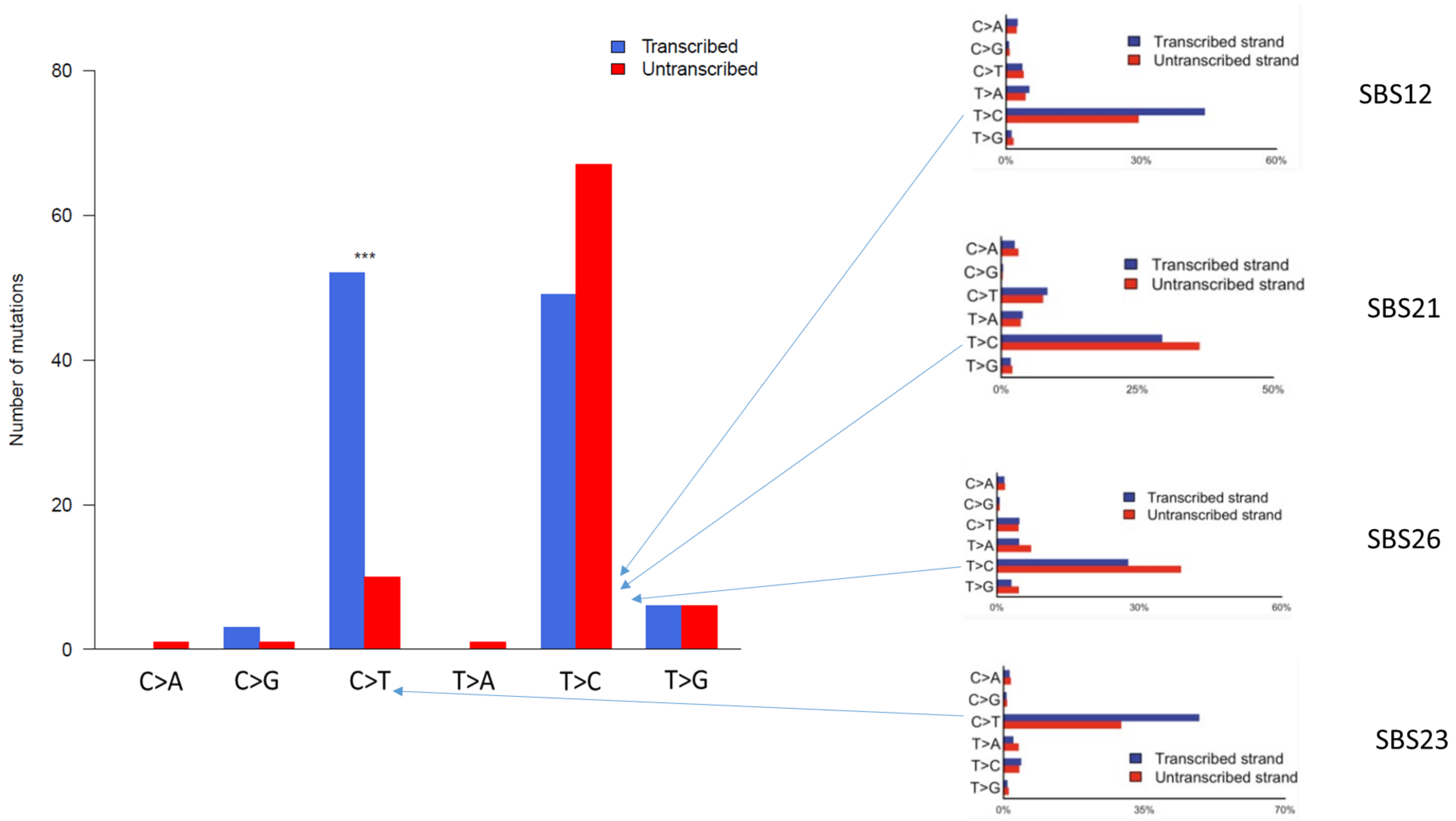

Supplementary Figure 2: Transcriptional strand bias contributed by various COSMIC mutational signatures extracted in 80 Myeloma XI primary tumours. Left panel: Number of substitutions observed on transcribed and untranscribed strand. Significant strand bias difference was assessed by proportion tests. ***: $P < 0.001$. Right panel: Screenshots of from COSMIC website (https://cancer.sanger.ac.uk/cosmic/signatures/SBS/) indicating transcriptional strand bias of COSMIC mutational signatures extracted from this study. COSMIC single base substitution (SBS) signatures 21 and 26 have opposing transcriptional strand bias with signature 12 for T>C.

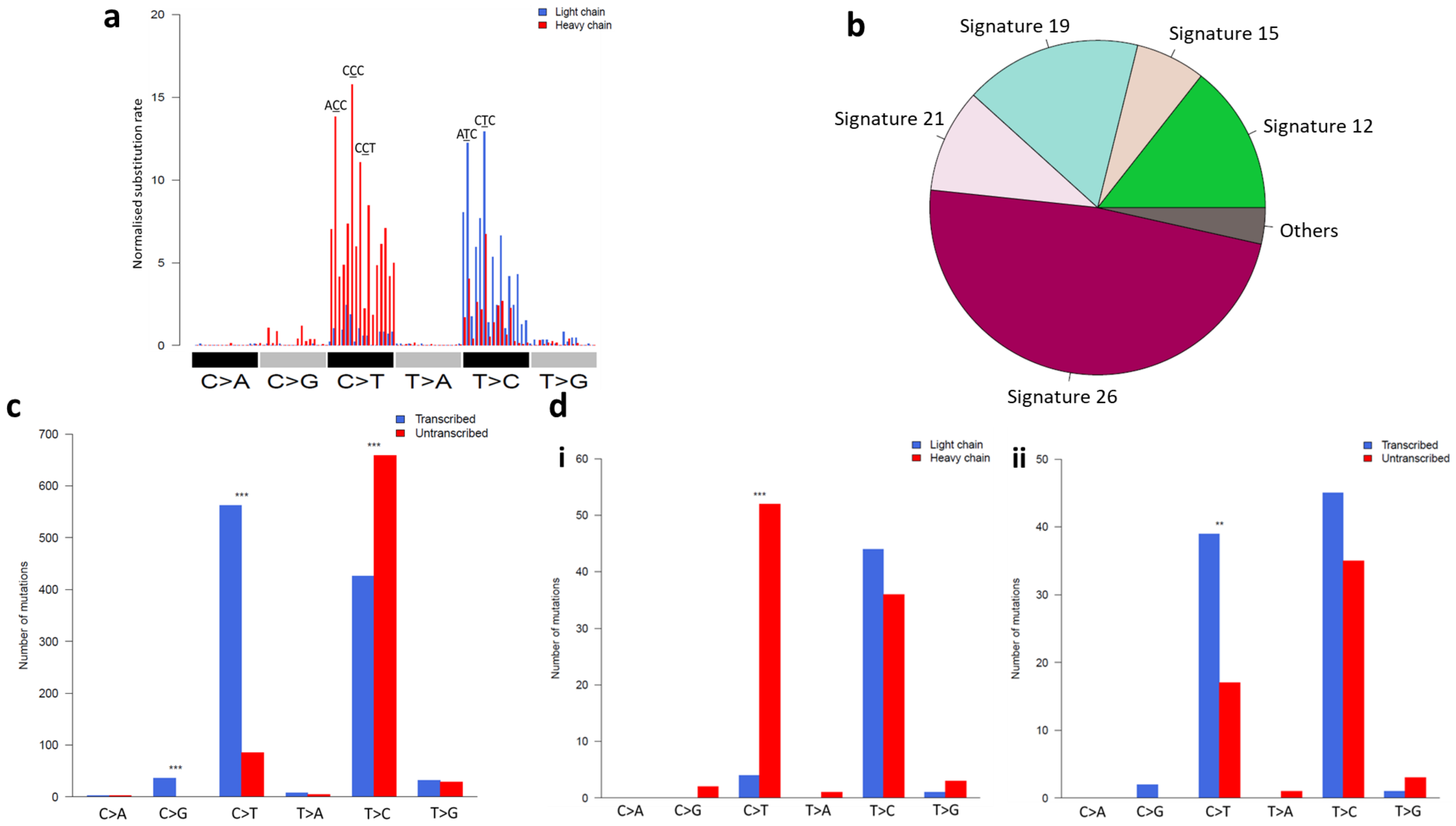
a
Light chain
Heavy chain
Normalised substitution rate
20
15
10
5
0
ACC
CCC
CCT
ATC
CTC
C>A
C>G
C>T
T>A
T>C
T>G
b
Signature 19
Signature 15
Signature 21
Signature 12
Others
Signature 26
c
Transcribed
Untranscribed
Number of mutations
700
600
500
400
300
200
100
0
***
***
***
C>A
C>G
C>T
T>A
T>C
T>G
d
i
Light chain
Heavy chain
Number of mutations
60
50
40
30
20
10
0
***
C>A
C>G
C>T
T>A
T>C
T>G
ii
Transcribed
Untranscribed
Number of mutations
50
40
30
20
10
0
**
C>A
C>G
C>T
T>A
T>C
T>G

Supplementary Figure 3: Mutational signatures in 850 tumours from CoMMpass dataset. (a) Mutational patterns by 96 trinucleotide context across all tumours**.** Substitution rate is normalised for trinucleotide context difference between mitochondrial light and heavy chains. (b) Contribution of COSMIC mutational signatures extracted by deconstructSigs. (c) Transcriptional strand biases across all mtDNA genes. (d) Replicative (i) and transcriptional (ii) strand bias for 22 tRNA genes. Significant difference in strand bias was assessed by proportion tests. **: $P < 0.01$, ***: $P < 0.001$.

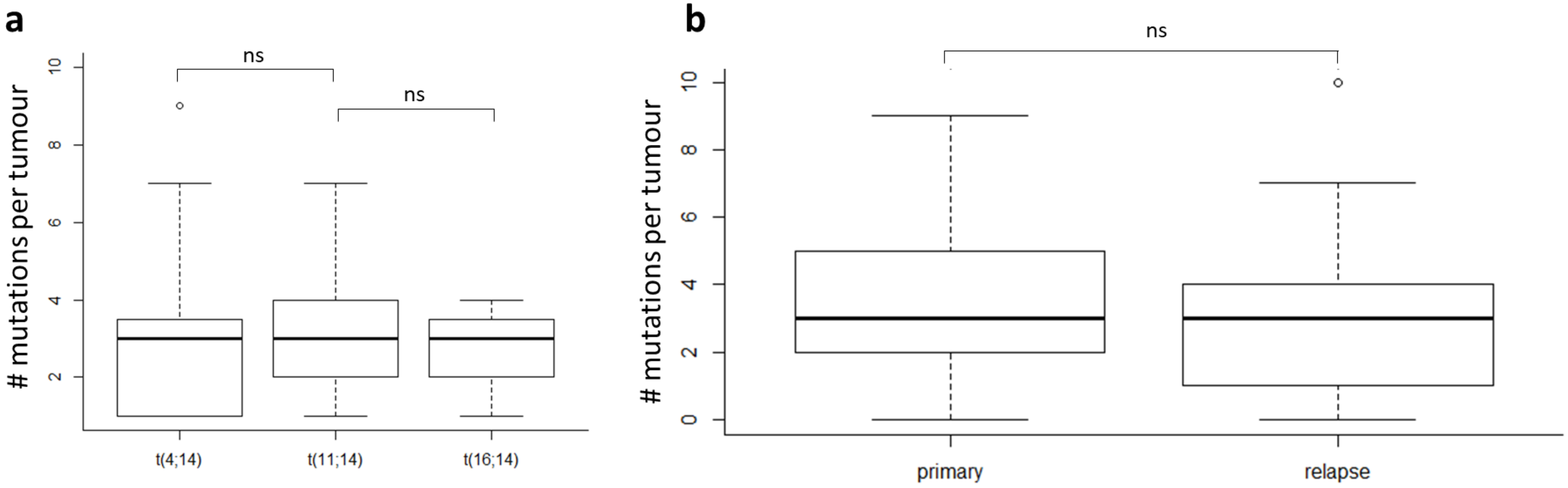

Supplementary Figure 4: Mitochondrial mutational burdens (a) across multiple myeloma subtypes and (b) between primary and relapsed tumours. Significant difference was assessed using Wilcoxon rank-sum test. Whisker bars extend within ± 1.5 × interquartile range. ns: not significant.

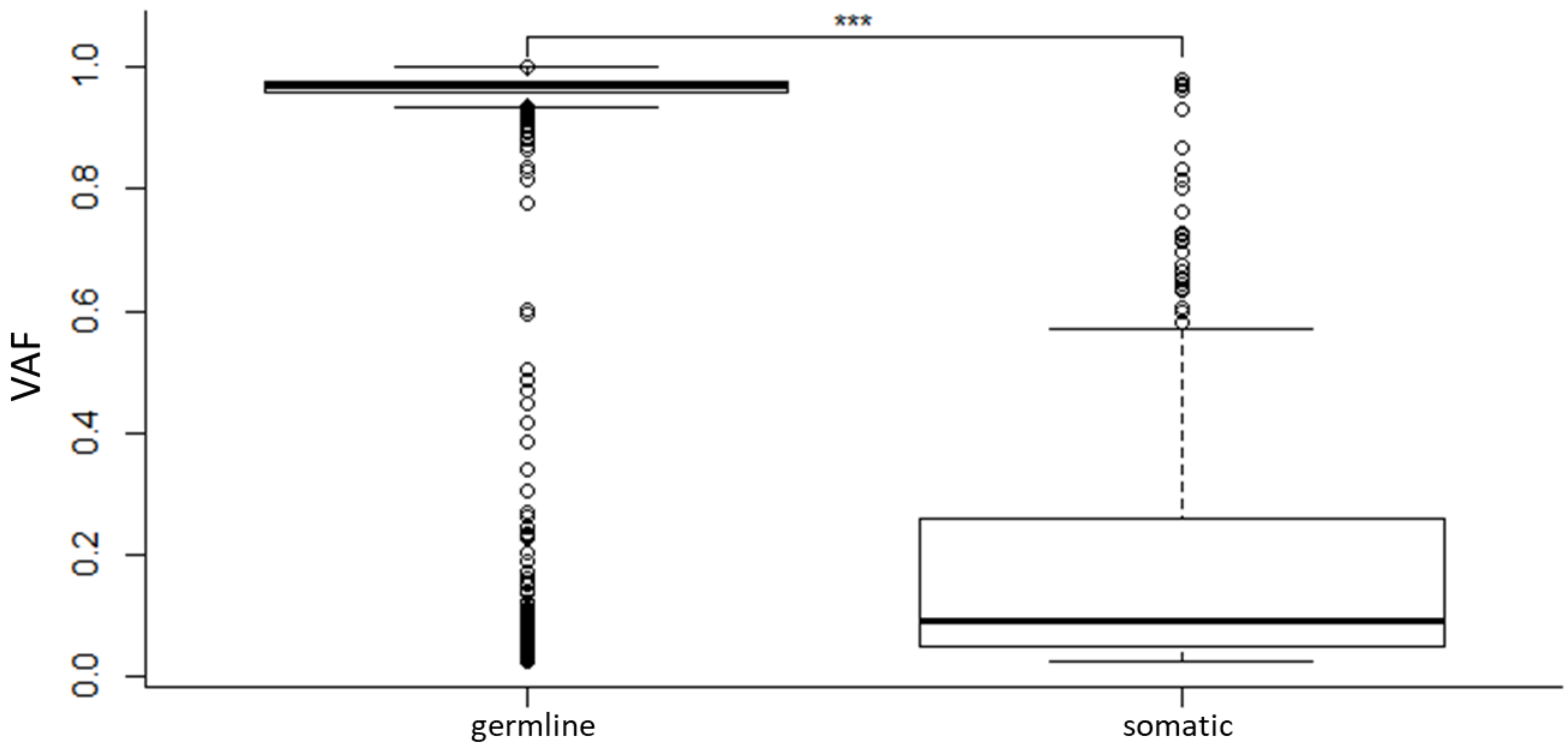

Supplementary Figure 5: Heteroplasmic level comparison between mitochondrial germline (n = 2137) and somatic mutations (n = 223). Significant difference was assessed using Wilcoxon rank-sum test. Whisker bars extend within $\pm$ 1.5 × interquartile range. ***: $P < 0.01$. VAF: variant allele frequency.

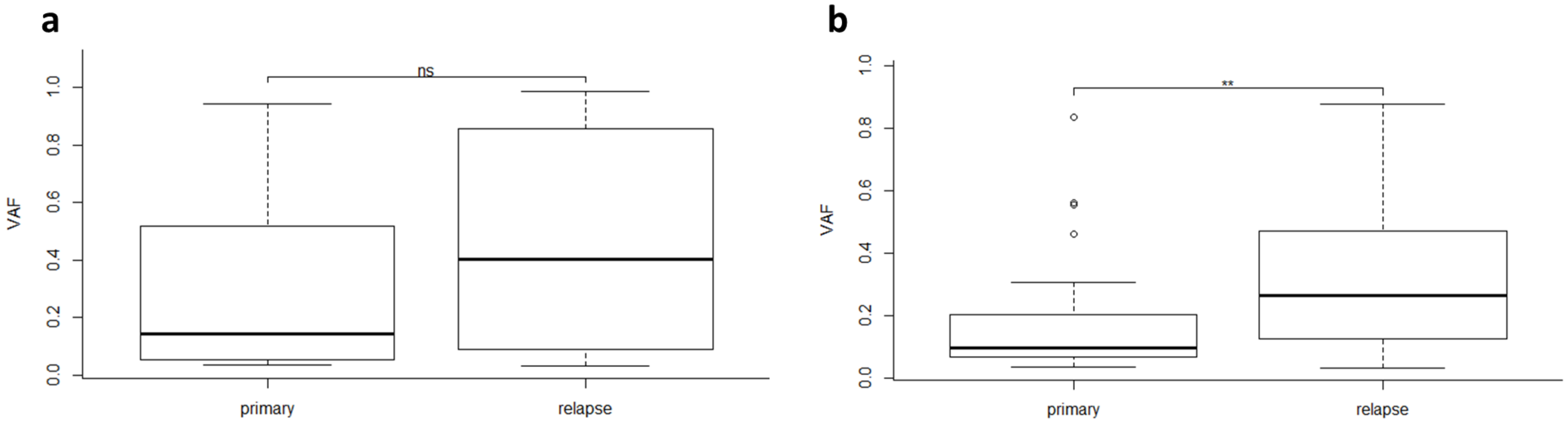

Supplementary Figure 6: Heteroplasmic level comparison between shared (a) silent mutations (n = 20) and (b) non-synonymous mutations (n = 47) in primary and matched relapse tumours. Significant different was assessed using paired Wilcoxon rank-sum test. Whisker bars extend within ± 1.5 × interquartile range. **: $P < 0.01$, ns: not significant. VAF: variant allele frequency.

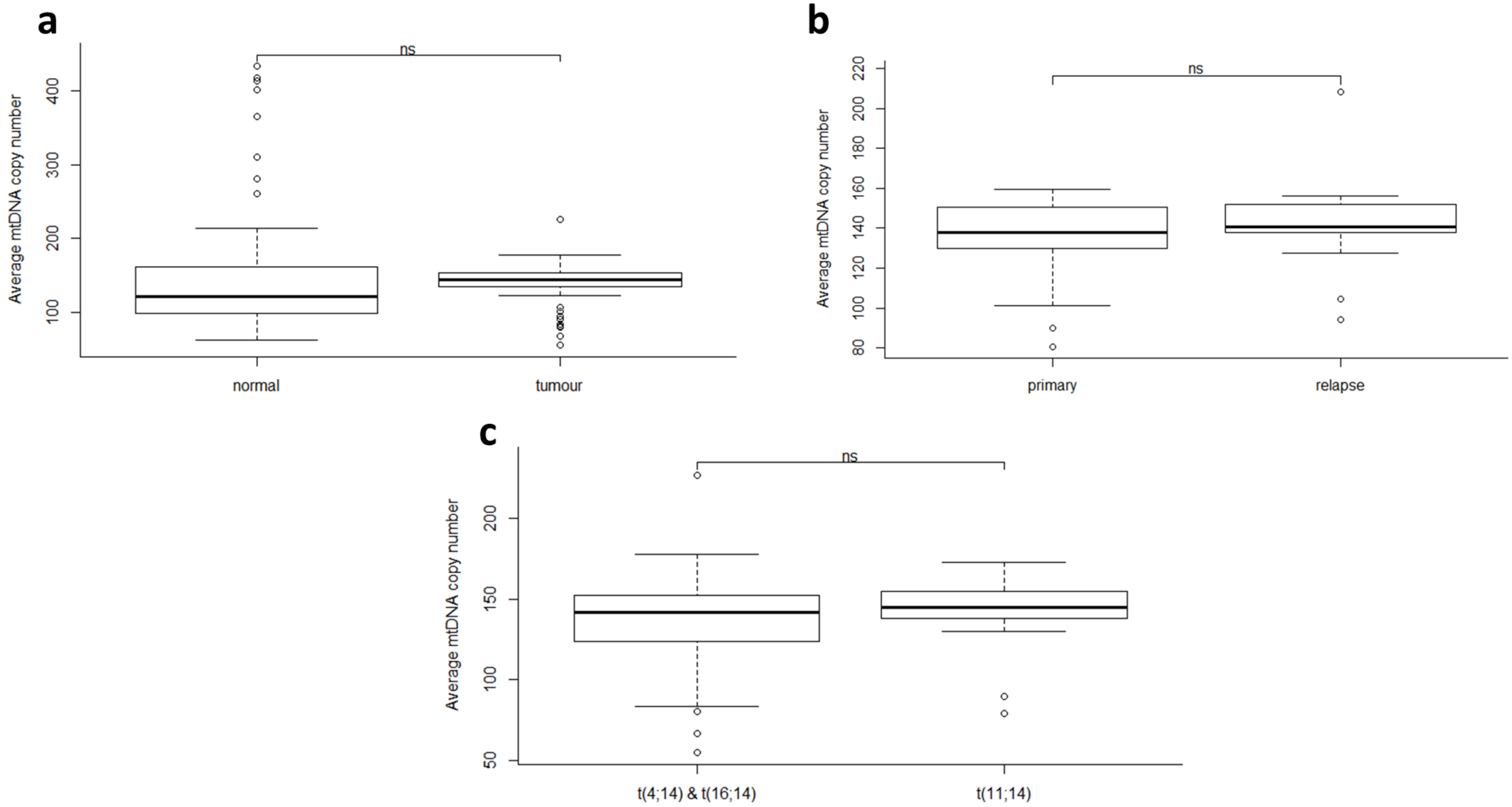

Supplementary Figure 7: Comparison of average mtDNA copy number between (a) normal and tumour, (b) primary and matched relapsed tumours, and (c) high-risk [t(4;14) and t(16;14)] and low-risk [t(11;14)] multiple myeloma subtypes. Significant different was assessed using paired Wilcoxon rank-sum test. Whisker bars extend within $\pm$ 1.5 × interquartile range. ns: not significant.
